# Active Components from *Cassia abbreviata* Prevent HIV-1 Entry by Distinct Mechanisms of Action

**DOI:** 10.3390/ijms22095052

**Published:** 2021-05-10

**Authors:** Yue Zheng, Xian-Wen Yang, Dominique Schols, Mattia Mori, Bruno Botta, Andy Chevigné, Martin Mulinge, André Steinmetz, Jean-Claude Schmit, Carole Seguin-Devaux

**Affiliations:** 1 Laboratory of Cellular and Molecular Oncology, Luxembourg Institute of Health, L-1445 Luxembourg, Luxembourg; yue.zheng@path.utah.edu (Y.Z.); yangxianwen@tio.org.cn (X.-W.Y.); andre.steinmetz@lih.lu (A.S.); 2 Laboratory of Virology and Chemotherapy, Department of Microbiology, Immunology and Transplantation, Rega Institute for Medical Research, KU Leuven, 3000 Leuven, Belgium; dominique.schols@kuleuven.be; 3 Department of Biotechnology, Chemistry and Pharmacy, University of Siena, 53100 Siena, Italy; mattia.mori@unisi.it; 4 Department of Chemistry and Technology of Drugs, Sapienza University of Rome, 00185 Rome, Italy; bruno.botta@uniroma1.it; 5 Department of Infection and Immunity, Luxembourg Institute of Health, L-4354 Esch-sur-Alzette, Luxembourg; andy.chevigne@lih.lu (A.C.); mmulinge@uonbi.ac.ke (M.M.); Jean-Claude.Schmit@ms.etat.lu (J.-C.S.); 6 Department of Biochemistry, School of Medicine, University of Nairobi, Nairobi, Kenya; 7 Service National of Infectious Diseases, Centre Hospitalier de Luxembourg, L-1210 Luxembourg, Luxembourg

**Keywords:** natural products, *Cassia abbreviata*, HIV-1 entry, piceatannol, structure-activity relationship, pharmacophoric studies, norartocarpetin

## Abstract

*Cassia abbreviata* is widely used in Sub-Saharan Africa for treating many diseases, including HIV-1 infection. We have recently described the chemical structures of 28 compounds isolated from an alcoholic crude extract of barks and roots of *C. abbreviata*, and showed that six bioactive compounds inhibit HIV-1 infection. In the present study, we demonstrate that the six compounds block HIV-1 entry into cells: oleanolic acid, palmitic acid, taxifolin, piceatannol, guibourtinidol-(4α→8)-epiafzelechin, and a novel compound named as cassiabrevone. We report, for the first time, that guibourtinidol-(4α→8)-epiafzelechin and cassiabrevone inhibit HIV-1 entry (IC_50_ of 42.47 µM and 30.96 µM, respectively), as well as that piceatannol interacts with cellular membranes. Piceatannol inhibits HIV-1 infection in a dual-chamber assay mimicking the female genital tract, as well as HSV infection, emphasizing its potential as a microbicide. Structure-activity relationships (SAR) showed that pharmacophoric groups of piceatannol are strictly required to inhibit HIV-1 entry. By a ligand-based in silico study, we speculated that piceatannol and norartocarpetin may have a very similar mechanism of action and efficacy because of the highly comparable pharmacophoric and 3D space, while guibourtinidol-(4α→8)-epiafzelechin and cassiabrevone may display a different mechanism. We finally show that cassiabrevone plays a major role of the crude extract of *CA* by blocking the binding activity of HIV-1 gp120 and CD4.

## 1. Introduction

Human Immunodeficiency Virus (HIV) infection was affecting about 38 million people (http://www.unaids.org/ accessed on 18 February 2021) around the world in 2019. The vast majority of HIV-infected patients live in low- and middle-income countries, particularly in Sub-Saharan Africa. Combined antiretroviral therapy (cART) has largely improved the life of HIV-infected patients [1], and is the leading factor in reducing the number of new HIV-infected cases worldwide. Current cART is, nevertheless, facing many challenges for life-long adherence [2]; treatment-experienced patients encountered viral mutants resistant to multiple drugs, and women are more prone to HIV infection than men in Sub-Saharan Africa [3]. The development of new anti-HIV drugs and potent microbicides is, therefore, highly required.

Around 30 antiretroviral drugs have been released for clinical practice, inhibiting HIV-1 at different stages of the viral life cycle [4]. Novel therapeutic strategies could take advantage of the multiple events involved in the entry process [5]. HIV-1 attaches to the cell membrane, engaging its surface envelope glycoprotein gp120 to bind subsequently the CD4 receptor and either to the C-X-C chemokine receptor type 4 (CXCR4) or C-C chemokine receptor type 5 (CCR5) co-receptor, and further triggering conformational changes in HIV-1 envelope glycoproteins leading to the membrane fusion process [6]. New compounds targeting interactions with cellular proteins/membranes or viral membranes might be less prone to selecting for drug resistance, should reduce the formation of viral reservoirs, and could additionally display broad spectrum antiviral effects than compounds targeting viral replication [7].

In the past decades, a high number of drugs have been developed from natural products and showed their critical power in medical therapies, such as the anti-malarial drug artemisinin and the anticancer drug paclitaxel [8]. The 2015 half Nobel Prize award for artemisinin’s discovery recently emphasized the enormous value of traditional medicine and ethnopharmacology. In this regard, *Cassia abbreviata* (*C. abbreviata*) is a tropical tree in *Cassia* genus, *Fabaceae* family, which is indigenous to South-East Africa, widespread in African countries and commonly used in the African local medicines [9]. Roots, barks, and leaves are taken as decoction or chewed, for healing abdominal pain, fever, cough, snake bite, malaria, infections, and, in particular, HIV-1 infection [10,11]. We have recently reported the isolation of 28 compounds from a crude extract of bark and roots of *C. abbreviata* [12]. Six bioactive compounds showed anti-HIV activity. Leteane et al. [13] has previously reported that a tanning free crude extract of *C. abbreviata* root inhibited HIV-1c (MJ4) replication in human peripheral blood mononuclear cells (PBMCs). It is known that a variety of compounds, including alkaloids, tannins, anthraquinones, and flavonoids, may contribute to the biological effect of plant medicines [10], while the active components of *C. abbreviata*’s, as well as their mode of action, are still unknown.

To better understand the mode of action of the traditional medicine plant *C. abbreviate*, we elucidated the antiviral activity of *C. abbreviata’s* crude extract (pulverized from barks and roots and extracted with 95% ethanol) against several HIV-1 strains, and demonstrated that six compounds isolated from *C. abbreviata were* inhibiting HIV-1 entry, including a novel compound cassiabrevone. We showed here the different mode of action of the active compounds in both structure studies and biological tests. Cassiabrevone plays a major role in the CE of *C. abbreviata* by inhibiting the binding activity of gp120 and CD4. Piceatannol blocks HIV-1 entry using a different mechanism by targeting cell and viral membrane. Optimized synthetic derivatives from cassiabrevone and piceatannol could be used as a microbicide.

## 2. Results

### 2.1. The Crude Extract and Purified Compounds of C. abbreviata Inhibit HIV-1 Entry

**Figure 1:**
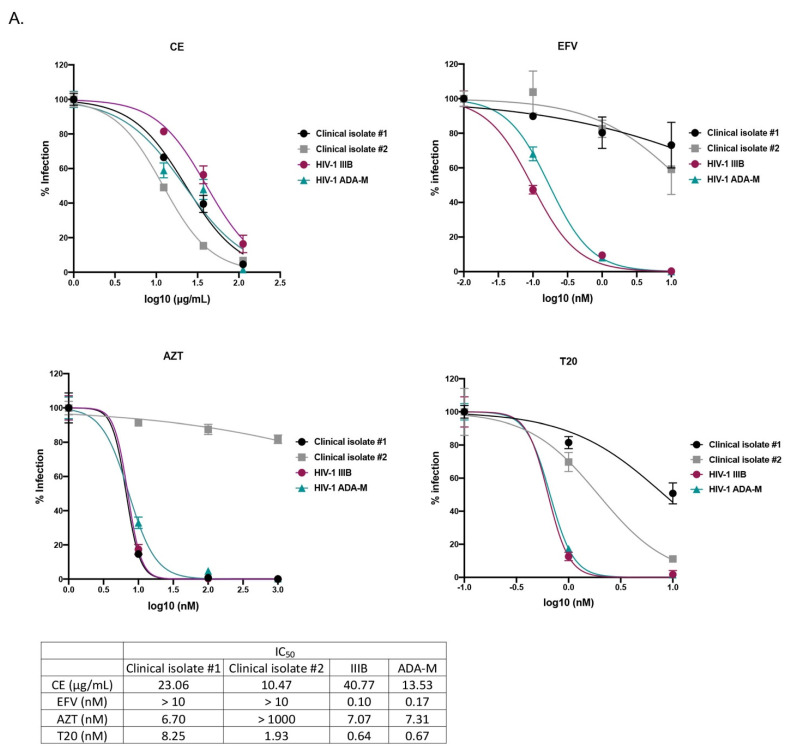

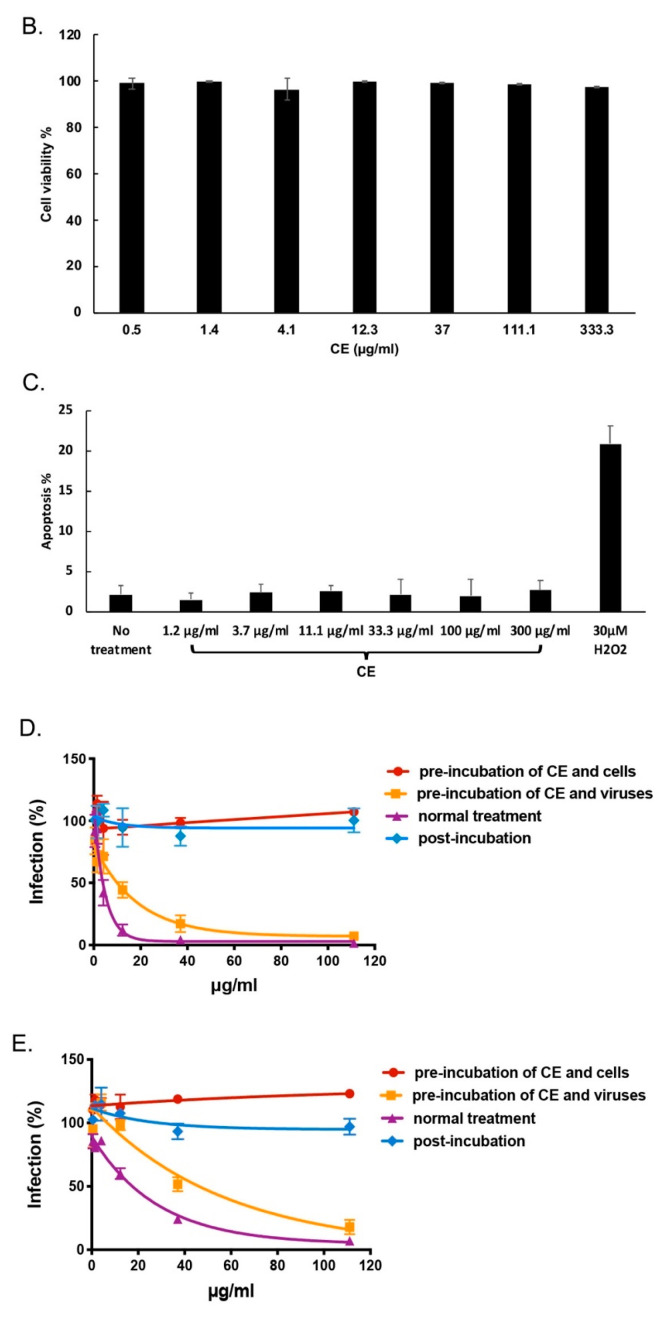
Crude extract (CE) of *C. abbreviata* inhibits HIV-1 entry into cells. (**A**) PBMCs isolated from healthy donor were treated with CE, or the NNRTI inhibitor efavirenz (EFV), the NRTI inhibitor azidothymidine (AZT) or the fusion inhibitor enfuvirtide (T20) for 7 days during infection. HIV-1 infection was assessed by measuring P24 in cell supernatants via ELISA. (**B**) PBMCs were treated with CE for 2 days. Cell viability was measured by flow cytometry. (**C**) PBMCs treated with CE were stained with Annexin-V/PI and measured by flow cytometry. Apoptosis level was calculated by counting both early apoptotic cells (Annexin-V+) and late apoptotic cells (PI+) (**D,E**). CE was tested in a multi-dosing time assay (**D**) using U373-CD4-CXCR4 cells against pseudotype virus pNL4.3Δ*Env*Luc-HXB2 and (**E**) using U373-CD4-CCR5 cells against pseudotype virus pNL4.3Δ*Env*Luc-BAL. CE was either pre-incubated with cells or viruses for 2 h, or incubated with cells and viruses during the 2 h spinoculation infection (normal treatment), or added to the cells after infection (post-incubation). HIV-1 infection was assessed by measuring luciferase in the cell supernatant. Three independent experiments were performed in triplicates.

The anti-HIV-1 activity of *C. abbreviata* was first assessed on MT4 cells and human peripheral blood mononuclear cells (PBMCs) from healthy donors using the HIV-1 reference strain IIIB (X4 tropic virus) and ADA-M (R5 tropic virus), as well as 2 non-B HIV-1 primary clinical isolates carrying several drug resistance mutations to nucleoside/nucleotide reverse transcriptase inhibitor (NRTI), to non-nucleoside reverse transcriptase inhibitor (NNRTI), and to protease inhibitors (PI). The crude extract (CE) inhibited HIV-1 infection in MT4 cells infected with the reference strain HIV-1-IIIB (X4 virus, IC50 = 21.75 ± 1.20 µg/mL) at non-toxic concentrations (CC50 above 1000 µg/mL). As shown in Figure 1A, CE inhibited HIV-1 infection in PBMCs with IC_50_ ranging from 10.47 to 40.77 µg/mL. The NNRTI efavirenz (EFV), the NRTI azidothymidine (AZT), and the fusion inhibitor enfuvirtide (T20) were used as positive controls. As expected, EFV and AZT did not inhibit viral infection of the respective clinical isolates which carried resistance mutations to NRTI and NNRTI, while T20 inhibited both HIV-1 reference strains and clinical isolates. We also examined the cytotoxicity of CE in PBMCs and found that CE was not toxic after 2 days treatment (Figure 1B). To further determine whether CE induces cell apoptosis, we measured the apoptotic cells by Annexin-V/PI staining after incubating CE and PBMCs for 48 h. As shown in Figure 1C, 30 µM H_2_O_2_, used as a positive control, induced 20% apoptosis, while CE did not induce any apoptosis as in non-treated cells. These data indicate that CE has an anti-HIV-1 activity without inducing any cytotoxicity or apoptosis.

To further characterize at which step of infection CE inhibited HIV-1, multi-dosing time assay experiments were performed with U373-CD4-CXCR4/CCR5 cells against infection of pseudotyped virus pNL4.3Δ*Env*Luc-HXB2 and pNL4.3Δ*Env*Luc-BAL.Pseudotyped viruses pNL4.3Δ*Env*Luc-HXB2/BAL are only able for one cycle of viral infection and allow us to assess the infection level by measuring the luciferase value in the cell supernatant. As shown in Figure 1D,E, the inhibitory effect of CE was apparent when CE was pre-incubated with the virus for 2 h cells (IC_50_ HXB2 = 2.57 µg/mL, IC_50_ BAL = 11.41 µg/mL) but not with the cells, as well as when CE was incubated with both cells and viruses during 2 h spinoculation infection (normal treatment) (IC_50_ HXB2 = 13.37 µg/mL, IC_50_ BAL = 67.40 µg/mL) but not after infection (CE was added during the first 2 h after the spinoculation). These results indicate that CE inhibits HIV-1 infection at an early stage of the HIV-1 infection independently of co-receptor usage

**Figure 2:**
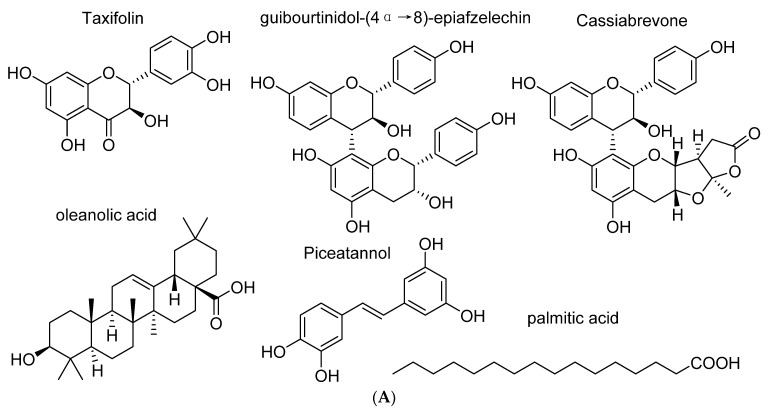

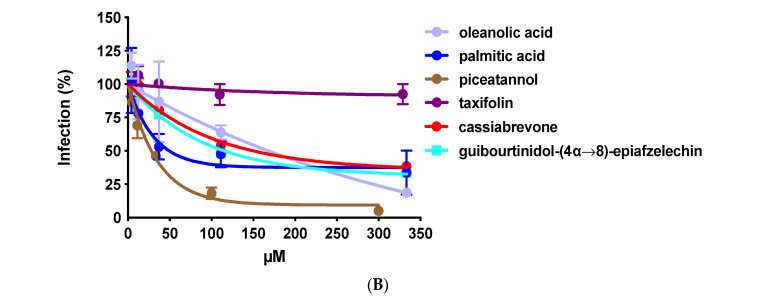
Anti-HIV-1 entry activity of oleanolic acid, palmitic acid, piceatannol, taxifolin, 4′7-dihydroxyflavan-(4,6)-3, 4′, 5,7-tetrahydroxyflavan and cassiabrevone. (**A**) Chemical structures of the compounds. (**B**) Oleanolic acid, palmitic acid, piceatannol, taxifolin, guibourtinidol-(4α→8)-epiafzelechin, and cassiabrevone were tested on U373-CD4-CXCR4 cells infected with pseudotype virus pNL4.3Δ*Env*Luc-HXB2 when added only at the time of infection. Percentage of infection versus the control cells infected without any compounds is represented.

Since we identified 6 compounds with an anti-HIV-1 activity [12], the anti-HIV activities of isolated compounds from *C. abbreviata* were further assessed against pseudotype viruses pNL4.3Δ*Env*Luc-HXB2 when the compounds were added only at the time of infection to identify the compounds targeting HIV-1 entry. Four known anti-HIV compounds were characterized: oleanolic acid, palmitic acid, taxifolin, and piceatannol (Figure 2A). In our experiment, oleanolic acid, palmitic acid, taxifolin and piceatannol inhibited HIV-1 entry at non-cytotoxic concentration showing IC_50_ at 34.87 ± 9.09 µM, 87.48 ± 16.12 µM, 240 ± 3 µM, and 10.28 ± 5.74 μM, respectively (Figure 2B). Moreover, we identified two other flavonoids that prevent HIV-1 entry: guibourtinidol-(4α→8)-epiafzelechin (IC_50_ 42.47 ± 3.88 µM) and a novel compound we named as cassiabrevone (IC_50_ 30.96 ± 5.02 µM). We will elucidate their mode of action in the last part of this work. We first focused our study on piceatannol since it was never described as an HIV-1 entry inhibitor.

### 2.2. Piceatannol Interacts with Both Cell and Viral Membranes and has a Synergistic Effect with HIV-1 Entry Inhibitors

**Figure 3:**
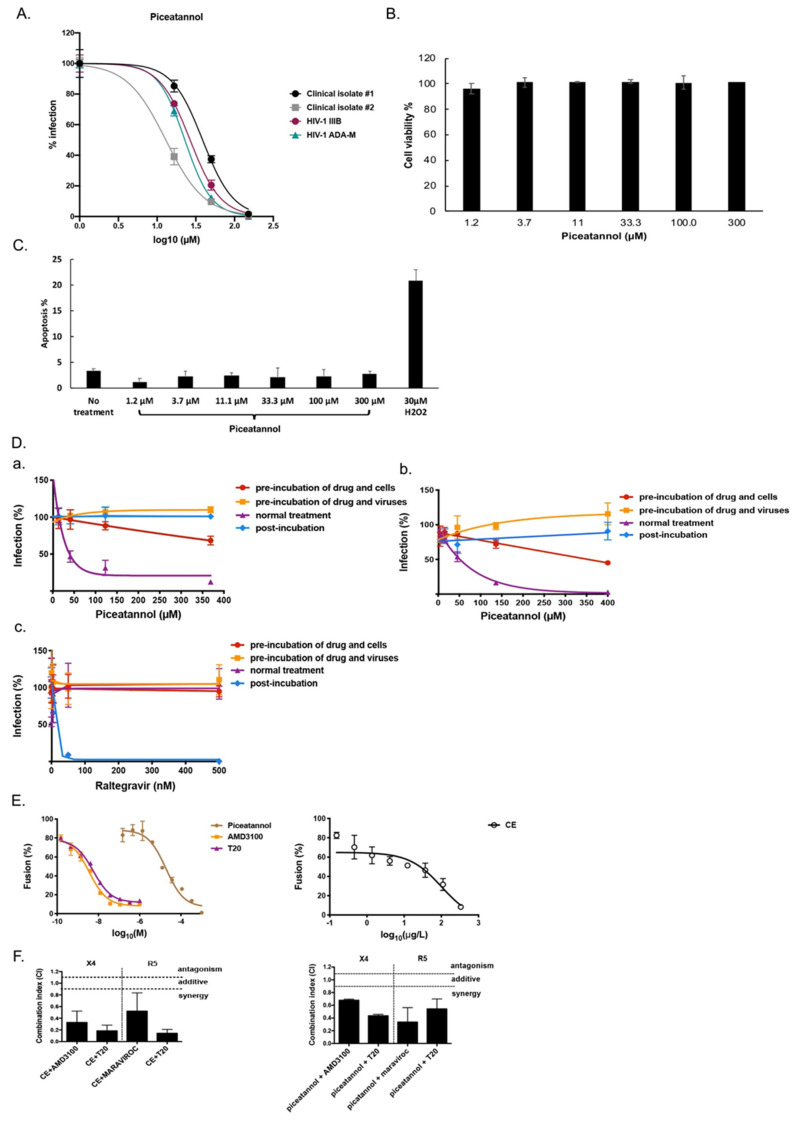
Piceatannol inhibits HIV-1 entry into cells. (**A**) PBMCs isolated from healthy donor were incubated with piceatannol during infection of HIV-1 reference strains and clinical isolates for 7 days. HIV-1 infection level was assessed by measuring P24 in cell supernatants via ELISA. (**B**) PBMCs were treated with piceatannol for 2 days. Cell viability was measured by flow cytometry. (**C**) PBMCs treated with piceatannol were stained with Annexin-V/PI and measured by flow cytometry. Apoptosis level was calculated by counting both early apoptotic cells (Annexin-V+) and late apoptotic cells (PI+). (**D**) Multi-dosing time assay with piceatannol was performed (a) using U373-CD4-CXCR4 cells against pseudotype virus pNL4.3Δ*Env*Luc-HXB2 and (b) using U373-CD4-CCR5 cells against pseudotype virus pNL4.3Δ*Env*Luc-BAL. (c) Raltegravir was tested in the multi-dosing time assay using U373-CD4-CXCR4 cells against pseudotype virus pNL4.3Δ*Env*Luc-HXB2. Percentage of infection relative to no drug treatment is represented. (**E**) Hela-ENV-Lai cells pretreated with piceatannol, CE, T20, or AMD3100 for 2 h were incubated with Hela-P4-CXCR4-LTRLacZ cells. HIV-1 fusion level was assessed by measuring β-Galactosidase activity. (**F**) Drug combination of CE or piceatannol and AMD3100/maraviroc/T20 was tested in U373-CD4-CXCR4/U373-CD4-CCR5 cells against pseudotype particles pNL4.3Δ*Env*Luc-HXB2/BAL. Combination Index (CI) at 95% maximal effective concentration (EC95) level was calculated using CompuSyn (ComboSyn, Paramus, NJ, USA).

We first confirmed that piceatannol protected PBMCs against HIV-1 IIIB and HIV-1 ADA-M (IC_50_ = 24.22 ± 7.13 µM and IC_50_ = 19.91 ± 0.22 µM, respectively), and against two non-B HIV-1 primary clinical isolates harboring multi-drug resistance to NRTI, NNRTI, and PI (IC_50_ of 37.72 ± 12.54 and 8.04 ± 3.07 µM) (Figure 3A). We also examined the cytotoxicity and apoptosis in the PBMCs treated with piceatannol for 2 days by flow cytometry. As shown in Figure 3B,C, piceatannol was not cytotoxic and did not induce any apoptosis. We next deciphered whether piceatannol targets cells or viruses using the multi-dosing time assay (Figure 3D). In contrast to CE, piceatannol inhibited HIV-1 infection at high concentration when it was pre-incubated with cells but not pre-incubated with the virus, indicating that piceatannol’s action is maximal when added together on both surface of cells and viruses. We did not find any effect of piceatannol when added after the step of infection in our pseudotyped assay although the integrase inhibitor raltegravir inhibited HIV-1 infection when added only after the time of infection.

To determine if CE and piceatannol block the HIV-1 fusion step, we treated Hela-ENV-Lai cells with CE, piceatannol, the CXCR4 inhibitor AMD100 or the fusion inhibitor T20 for 2 h and then co-cultured with Hela-P4-CXCR4LTRLacZ cells for 2 days. We can assess the effect of CE and piceatannol on the fusion process by measuring the β-galactosidase activity when the fusion step occurs between Hela-P4-CXCR4 cell and Hela-ENV-Lai cell. As shown in Figure 3E, both CE and piceatannol inhibit HIV-1 fusion efficiently with an IC_50_ of 22 µM and 33.35 µg/mL, respectively.

To further investigate if CE and piceatannol have a synergistic effect with known inhibitors of the fusion step, including the fusion inhibitor T20, the CXCR4 inhibitor AMD3100, or the CCR5 inhibitor Maraviroc, we treated U373-CD4-CXCR4/U373-CD4-CCR5 cells with single drug or combined drugs during the infection of pseudotyped viruses pNL4.3Δ*Env*Luc-HXB2/pNL4.3Δ*Env*Luc-BAL for 2 h. By calculating the combination index (CI) using the qualitative Chou and Talalay’s method [14], both CE and piceatannol showed synergistic effect with the three known inhibitors T20, AMD100 and Maraviroc (CI < 0.9), which indicates that CE and piceatannol employ a different mechanism to prevent HIV-1 entry than the fusion inhibitor T20 or the CXCR4/CCR5 inhibitors.

### 2.3. CE of C. abbreviata Affects gp120/CD4 Binding Whereas Piceatannol Interacts with Cellular Membranes

**Figure 4:**
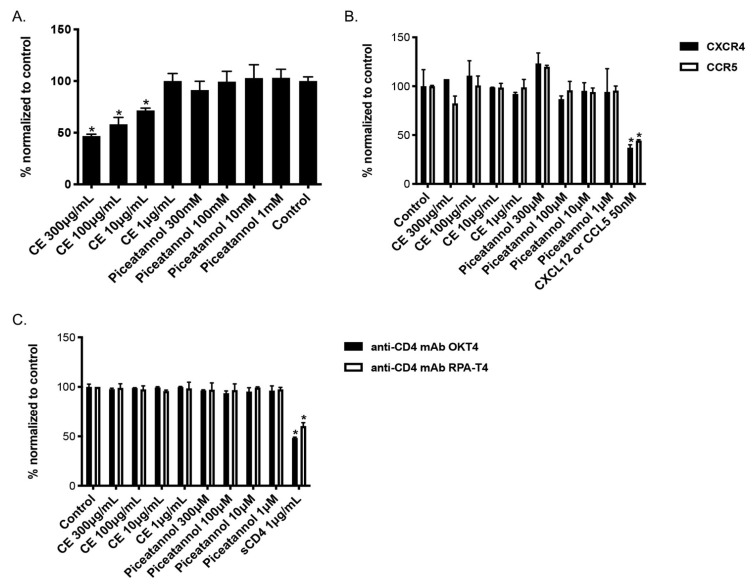
Binding activity of *C. abbreviata*’s crude extract and piceatannol. (**A**)**.** CE and piceatannol were tested in an in-house ELISA assay against gp120 binding activity. Data were normalized to control cells without CE/piceatannol treatment. (**B**) U373-CD4-CXCR4/CCR5 cells were incubated with either anti-CD4 antibody OKT4 or RPA-T4 in the presence of CE and piceatannol. Soluble CD4 (sCD4) was used as a positive control for binding anti-CD4 antibodies. (**C**) U373-CD4-CXCR4/CCR5 cells were incubated with either anti-CXCR4 antibody 12G5 or anti-CCR5 antibody 2D7 in the presence of CE and piceatannol. The chemokines CXCL12 and CCL5 were used as positive controls for binding CXCR4 and CCR5, respectively. Data were normalized to the MFI of the control cells. Three independent assays were performed for each binding assay, and ANOVA analysis was done for statistical significance. * *p* < 0.05.

To further examine whether CE and piceatannol affects the binding activity of HIV-1 gp120 on the host CD4 receptor, we performed an ELISA assay to analyze if CE or piceatannol could compete with gp120 to bind CD4. As shown in Figure 4A, CE blocked the binding between CD4 and gp120 in a dose-dependent manner, while piceatannol did not. We then further tested if CE and piceatannol inhibited CD4-gp120 binding by targeting CD4. The binding activity of two anti-CD4 monoclonal antibodies targeting the CD4 domain 1 and 3, in the presence or absence of CE and piceatannol was assessed. As shown in Figure 4B, the positive control soluble CD4 (sCD4) inhibited the two anti-CD4 antibodies binding to CD4, while neither CE nor piceatannol did. Thus, CE inhibits CD4-gp120 binding by targeting gp120.

We observed in Figure 1 and Figure 3 that CE and piceatannol inhibited viral infection of both X4 and R5 viruses, suggesting no specific effect of CE or piceatannol on the co-receptors CXCR4 and CCR5. We next measured CXCR4/CCR5 binding with their respective chemokine in the presence or absence of CE and piceatannol. As shown in Figure 4C, the chemokines CXCL12 (C-X-C motif chemokine ligand 12) and CCL5 (C-C motif chemokine ligand 5) inhibited CXCR4/CCR5 binding, respectively, while neither CE nor piceatannol did.

In line with this evidence, piceatannol, but not CE, inhibited the infection of pseudotype particles of vesicular stomatitis virus (VSV) G proteins (IC_50_ = 79.23 ± 17.20 μM, Table 1), indicating that piceatannol inhibits HIV-1 entry independently of any receptors. To determine if CE or piceatannol had a broad-spectrum antiviral activity or was specific to HIV-1, we tested both on diverse viruses. CE and piceatannol demonstrated anti-viral activity on simplex virus infection HSV-1 and HSV-2 (IC_50_ around 45.0 μM) but not on influenza, para-influenza, HCV, coxsackie virus, RSV, reovirus, sindbisvirus, punta toro virus, yellow river virus, feline corona virus, and feline herpes virus (Table 1).

### 2.4. Microbicide Activity of Piceatannol

**Figure 5:**
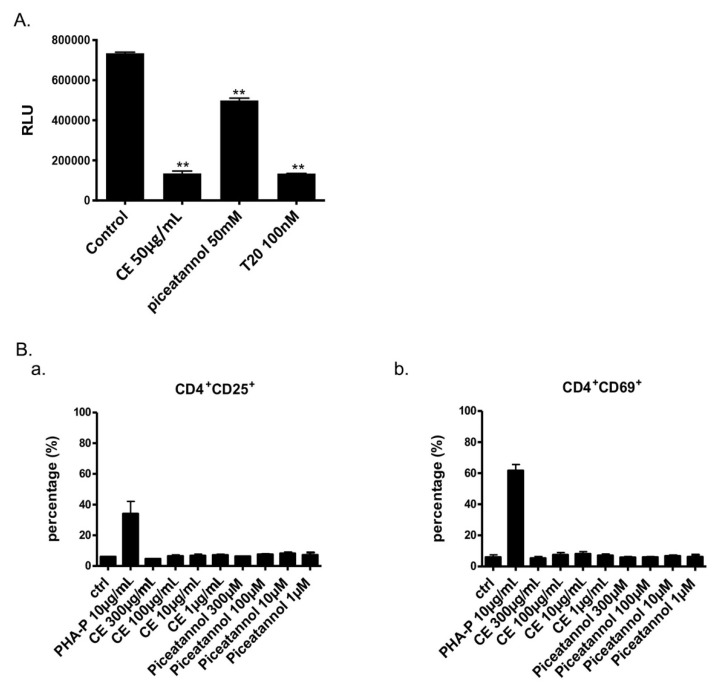
Evaluation of Piceatannol as a microbicide. (**A**) CE, piceatannol and T20 were tested in a dual chamber system. Compounds and HIV-1 ADA-M viruses were added to Hela cells in the upper chamber. Luciferase of TZM-bl cells in the lower chamber was measured at 24 h post-infection. (**B**) Expressions of CD25 (a) and CD69 (b) on PBMCs with or without CE and piceatannol treatment were measured by flow cytometry. ANOVA analysis was performed for statistical significance. ** *p* < 0.01.

Since both CE and piceatannol were active against HIV-1, HSV-1, and HSV-2, we further checked its potential as a microbicide. We next tested piceatannol in a dual-chamber system mimicking the epithelium female genital tract (Figure 5A). Both CE and piceatannol inhibited HIV-1 infection of TZM-Bl cells without affecting the confluence layer’s TEER and epithelial cells viability. At their IC_50_ concentration, CE decreased by more than 4 times HIV-1 infection (from 100 to 18.41%), while piceatannol diminished it by 33% (from 100 to 66.9%). For potential microbicide application, anti-HIV compounds should not stimulate target cells. PHA increased the expression of CD25 (early activation marker) and CD69 (late activation marker), whereas CE and piceatannol did not have any effect (Figure 5B). Finally, CE and piceatannol did not prevent transmission of DC-SIGN (Dendritic Cell-Specific Intercellular adhesion molecule-3-Grabbing Non-integrin)-captured virus to CD4^+^ T cells in vitro (data not shown).

### 2.5. Structure-Activity Relationship of Piceatannol

**Figure 6:**
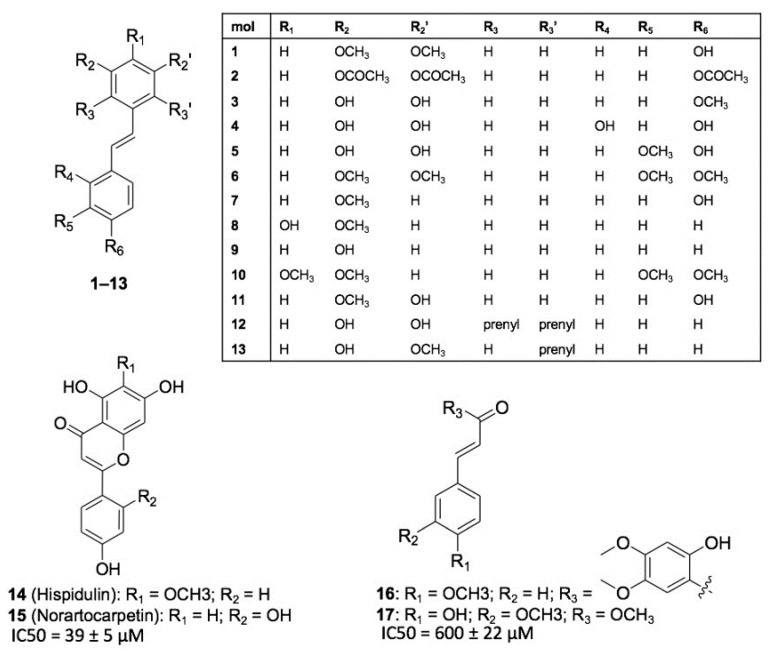
SAR of piceatannol. The chemical structure of compounds **1**–**17** used to afford SAR of piceatannol is shown. Molecules are grouped based on their chemical structures: (i) stilbene derivatives **1**–**13**; (ii) flavones **14** and **15**; (iii) styrene derivatives **16** and **17**. IC_50_ values of the compounds are mentioned when the compound displayed anti-HIV-1 activity.

Piceatannol emerged from this study as a promising anti-HIV-1 compounds extracted from *C. abbreviata*. Since molecular details of piceatannol interaction with its target(s) are currently not available, we performed a structure-activity relationship (SAR) study with the aim to understand the chemical and pharmacophoric features of piceatannol that are relevant for its anti-HIV activity. Seventeen chemical analogues of piceatannol (molecules **1**–**17**, Figure 6) were designed and retrieved from commercial vendors and from an in-house library of natural products and their derivatives [15,16,17,18,19] and tested in vitro against HIV-1 entry and HIV-1 infection using the pseudotype and the MTT assay, respectively. Compounds **1**–**13** are stilbene derivatives endowed with subtle chemical modifications with respect to piceatannol (i.e., molecules **4** and **5**) or bearing larger substituents (i.e., **6**–**11** and longistilines **12** and **13**) showing no anti-HIV activity. Besides, the two flavones hispidulin **14** and norartocarpetin **15**, and the two styrene derivatives **16** and methyl ferulate **17** were tested. Only compounds that do not bear the stilbene scaffold showed some activity: methyl ferulate **17** at high concentrations (IC_50_ = 600 ± 22 μM) and norartocarpetin **15** (IC_50_ = 39 ±5 μM, CC_50_ > 500 μM), the latest showing antiretroviral activity similar to piceatannol. Norartocarpetin is structurally related to taxifolin, although it is noticeably more potent. This result clearly indicates that pharmacophoric features of piceatannol are highly specific, and that subtle modifications to its chemical structure determine a significant drop of anti-HIV-1 activity. Accordingly, it is not surprising that stilbene derivatives bearing larger substitutions, such as molecules **1**, **2**, **6**–**11**, and longistilines **12** and **13** proved inactive, as well. Similar to piceatannol, the comparison between structure and activity of **14** and **15** further highlights the key role of polyphenols in providing anti-HIV activity.

### 2.6. Mode of Action of the Two Flavonoids Inhibiting HIV-1 Entry

**Figure 7:**
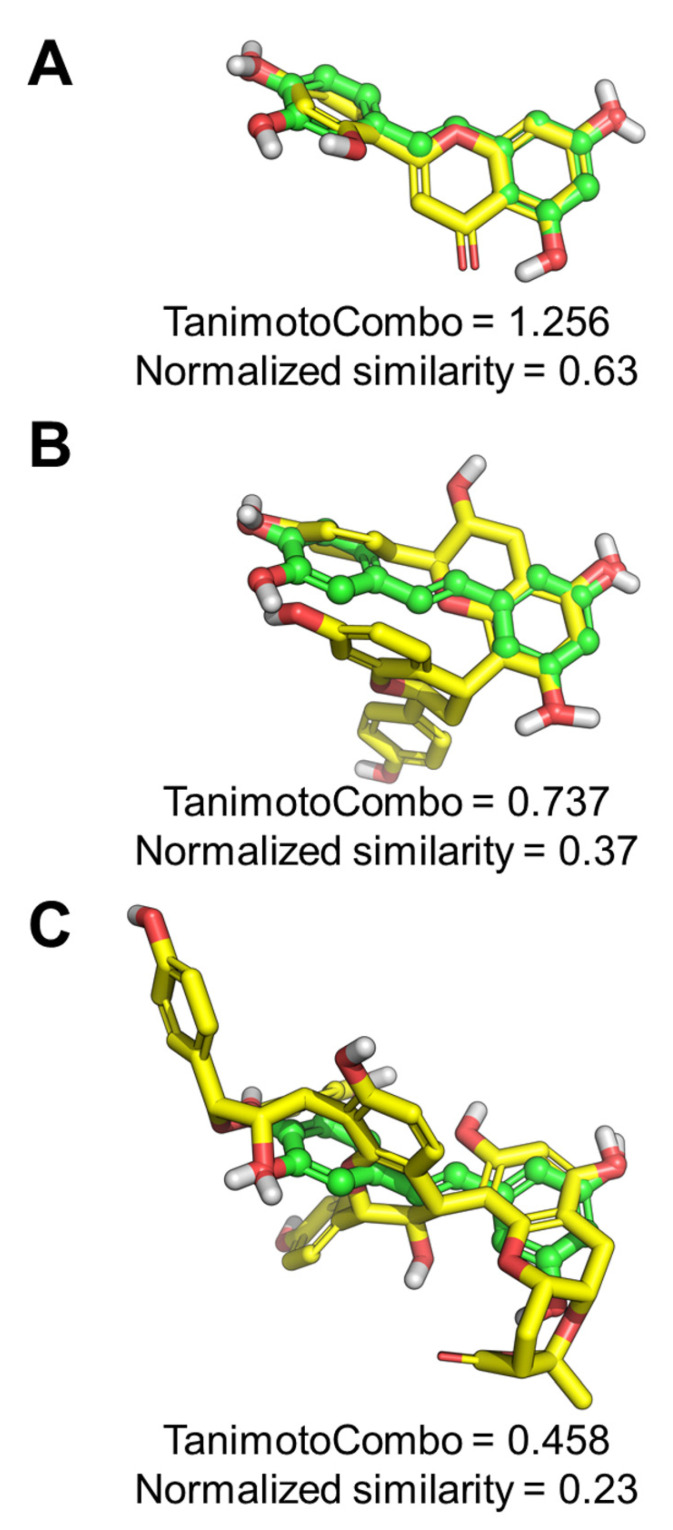
Pharmacophoric studies. (**A**) ROCS alignment between the query built on piceatannol and norartocarpetin, 4′7-Dihydroxyflavan (4,6)-3, 4´, 5,7- tetrahydroxyflavan (**B**), and cassiabrevone (**C**). Piceatannol is shown as green balls and sticks with the same orientation in the three panels. Aligned ligands are shown as yellow sticks. Non-polar H atoms were omitted. The TanimotoCombo and normalized similarity scores are reported for each pair of compounds.

To develop the mode of action, we first performed a ligand-based studies to provide insights into the mechanism of action of piceatannol, norartocarpetin, cassiabrevone, and 4′7-Dihydroxyflavan (4,6)-3, 4’, 5,7- tetrahydroxyflavan using the Rapid Overlay of Chemical Structures (ROCS) program (OpenEye Scientific Software). As shown in Figure 7A, a normalized similarity of 0.63 was obtained by comparing piceatannol to norartocarpetin, suggesting that these compounds cover a highly similar pharmacophoric and 3D space. In contrast, 4′7-dihydroxyflavan (4,6)-3, 4´, 5,7- tetrahydroxyflavan and cassiabrevone share a moderate similarity with piceatannol, (normalized similarity = 0.37 and 0.23, respectively, Figure 7B–C), which suggests a different mechanism of action for these molecules compared to piceatannol and norartocarpetin.

**Figure 8:**
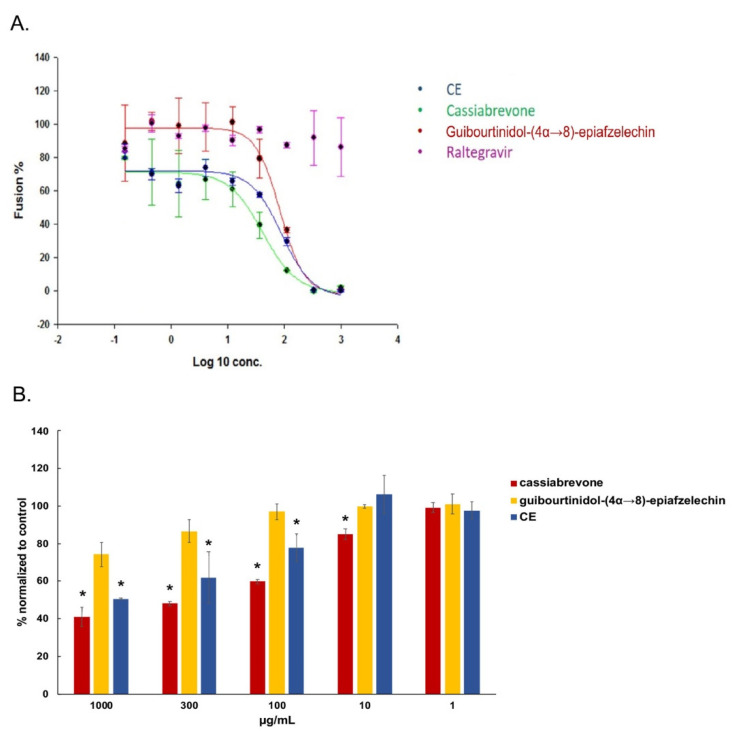
Cassiabrevone inhibits gp120/CD4 interaction. (**A**) Hela-P4-CXCR4 cells pretreated with CE, guibourtinidol-(4α→8)-epiafzelechin), and cassiabrevone were incubated with hela-ENV-Lai cells. β-Galactosidase activity was accessed. Data were normalized to control cells without any drugs to give the percentage of fusion. (**B**) Cassiabrevone and guibourtinidol-(4α→8)- -epiafzelechin were tested in an in-house ELISA assay against gp120 binding activity. Data were normalized to control cells without compound treatment. ANOVA analysis was performed for statistical significance. * *p* < 0.05.

For further validation, we then tested cassiabrevone and guibourtinidol-(4α→8)-epiafzelechin in a fusion assay and showed that both two compounds inhibited viral fusion similarly to CE (Figure 8A). Moreover, cassiabrevone but not guibourtinidol-(4α→8)-epiafzelechin inhibited significantly gp120/CD4 binding activity in a dose-dependent manner as observed for CE (Figure 8B), indicating that cassiabrevone may play the major role of the crude extract of *C. abbreviata* and display different mode of action from piceatannol (Figure 3D).

Finally, taken together, our studies confirmed that the traditional medicine plant *C. abbreviata* displayed anti-HIV-1 activity and identified six active components that inhibits HIV-1 entry. Importantly, we revealed a novel compound cassiabrevone and showed that cassiabrevone played the major role of the crude extract of *C. abbreviata*. Moreover, we delineated the anti-HIV mechanism of piceatannol and showed its potential as a microbicide.

## 3. Discussion

Many antiviral agents have been identified from plant sources but their mechanisms of action are poorly understood. *C. abbreviata* is indigenous to Africa, and commonly used against infectious diseases [9,11]. Nevertheless, *C. abbreviata*’s bioactive compounds were not characterized. The anti-HIV-1 activity of a *C. abbreviata*’s ethanol extract was first described by Leteane’s group [13]. In our study, we have performed a secondary ethyl acetate partition after a primary ethanol extraction to obtain a crude extract (CE) with enhanced anti-HIV-1 activity and lower cytotoxicity. We sought here (i) to determine at which step of infection the CE of *C. abbreviata* inhibited HIV-1, (ii) to screen active compounds against HIV-1 entry, and (iii) to unravel the mechanism of action of isolated compounds preventing HIV-1 entry.

Our results indicate that CE inhibited HIV-1 infection at an early stage of the HIV-1 entry process independent of co-receptor usage and interacted with HIV-1 gp120. By testing compounds only at the time of infection against pseudotyped viruses, we found that oleanolic acid, palmitic acid, taxifolin, and piceatannol inhibited HIV-1 entry at the micromolar range for non-cytotoxic concentrations. These results are consistent with previous studies showing that palmitic acid and oleanolic acid blocked gp120-CD4 interaction [20,21] and that oleanolic acid, piceatannol and taxifolin inhibited HIV-1 infection [22,23,24,25]. According to our findings, the major active components of CE inhibited the interaction between gp120 and CD4, and, especially, cassiabrevone, a flavonoid with a new structure purified from CE, reproduced this feature. CD4 and co-receptor binding sites, as well as variable loops and glycans of gp120, were already proposed as therapeutic targets. For instance, several small-molecule attachment inhibitors targeting the conserved CD4 binding region within gp120 have been described [26,27,28,29]. Fostemsavir, a prodrug of the HIV-1 attachment inhibitor temsavir, was recently approved in clinic for heavily treatment-experienced HIV-1 patients. Using homology models, fostemsavir was proposed to bind to the unliganded conformation of gp120 within the structurally conserved outer domain adjacent to the CD4 binding loop. It is tempting to speculate that other compounds derived from natural products may display similar activity on gp120 conformation or binding with CD4. In our hands, cassiabrevone may display such potential and might explain the main effects of *C. abbreviata* against HIV-1 entry.

In this study, we have shown, for the first time, that piceatannol inhibits HIV-1 entry and focused our work to explain its mode of action, as well as its potency as microbicide and against other viruses. Clouser et al. have reported that piceatannol inhibited HIV-1 replication with an IC_50_ of 21.4 µM in accordance with our results [24]. In our hands, time dose-dependent experiments and fusion assays did not support piceatannol as an HIV-1 integrase inhibitor as previously reported in non-cell-based assays [25,30]. We did not find any effect of piceatannol when added after the step of infection in our pseudotype assay although the integrase inhibitor raltegravir inhibited HIV-1 infection when added only after the time of infection (Figure 3). Taking into consideration that piceatannol did not affect neither CD4 and CCR5/CXCR4 binding, nor the interaction between CD4 and gp120, as well as the fusion process, we assume that piceatannol may interact with virus attachment by adsorbing at either the cell surface or the virus surface. Piceatannol also showed a synergistic effect with both co-receptor and fusion inhibitors, which indicates that piceatannol employs a different mechanism to prevent HIV entry. This hypothesis is reinforced by the observation that piceatannol inhibited VSV infection since VSV enters into target cells by endocytosis and not by interfering with a specific cellular receptor. From a chemical standpoint, piceatannol (trans-3,3′,4,5′-tetrahydroxystilbene) is a natural analogue of resveratrol (trans-3,4′,5′-trihydroxystilbene). Many analogues of resveratrol have been reported, as well, to inhibit HIV-1 infection [31], and trans-3,3′,4,4′,5,5′-hexahydroxy-stilbene was shown recently to inhibit HIV-1 entry before the fusion step [32]. Both piceatannol and 3,3′,4,4′,5,5′-hexahydroxy-trans-stilbene display better anti-HIV activity than resveratrol, suggesting that the additional hydroxyl groups to the basic stilbene rings may increase the anti-HIV activity by strengthening compound affinity or by facilitating its action on the membrane surface. Furthermore curcumin, having two phenols connected by a carbon chain, has structural similarity to piceatannol and can affect viral membrane fluidity to block viral entry [33] similarly to rigid amphipathic fusion inhibitors (RAFIs) and LJ001, which affects the membrane of HIV [7,34]. Interestingly, resveratrol was also shown to inhibit simplex virus vaginal infection in a mouse model [35].

The SAR and ligand-based studies highlighted the highly specific chemical and pharmacophoric features of piceatannol that are required to exert anti-HIV-1 activity. Moreover, the flavone norartocarpetin was identified as a conformational restrained analogue of piceatannol endowed with comparable efficacy. Piceatannol and norartocarpetin share a number of pharmacophoric and shape similarities, indicating a common mechanism of action. In contrast, 4′7-dihydroxyflavan (4,6)-3, 4´, 5,7- tetrahydroxyflavan and cassiabrevone share only a moderate similarity with piceatannol, which suggests that these molecules might inhibit HIV-1 replication through a mechanism that is different from that observed for piceatannol itself and norartocarpetin. The SAR study also emphasized the key role of polyphenols in providing anti-HIV activity, in agreement with recent reports [32,36,37]. One may note that piceatannol has a catechol moiety, widespread in natural products, which is considered as a protein-reactive species [38,39]. However, the anti-HIV-1 effect of piceatannol was found in this work to be highly specific by targeting only the entry step of the envelope virus HIV, HSV, and VSV without significant cytotoxicity.

Importantly, we have shown that both CE and piceatannol inhibited HIV infection in an in vitro dual-chamber model, mimicking the epithelium of the female genital tract [35], suggesting that the active components can cross the epithelial barrier without any toxicity on epithelial cells and no further activation of PBMCs. These data indicate the potential of piceatannol or cassiabrevone to be used as a lead structure for microbicides, although it did not prevent cell-to-cell and DC-SIGN-mediated viral transmission in vitro. In addition, the dual HIV/HSV activity of piceatannol could be crucial for microbicide applications since genital HSV-2 infection has been shown to potentiate HIV transmission and infection [40].

## 4. Conclusions

There is still an ongoing need for new potent classes of antiretroviral drugs with improved safety and tolerability profiles to sustain long-life antiretroviral therapy. In the present work, we have shown that 6 active components block HIV-1 entry. Importantly, we have isolated a novel flavonoid named as cassiabrevone and identified cassiabrevone as one of the active components from *C. abbreviata* that prevents HIV-1 entry by targeting gp120. Similar to piceatannol, cassiabrevone might have the potential to be used as a microbicide. Optimized synthetic derivatives from cassiabrevone and/or piceatannol should be resolved to reach a future therapeutic efficacy in humans.

## 5. Materials and Methods

### 5.1. Plant Extraction and Compounds Purification

Barks and roots of *C. abbreviata* were collected from mature shrubs in Makueni County, Kenya. Its identity was confirmed by DNA barcoding approach in the lab. All materials were pulverized before extraction. The crude extract (CE) was obtained through a first extraction of barks and roots from *C.* *abbreviata* that were pulverized with 95% ethanol, and a second extraction with ethyl acetate, and dried. The extracts were combined and concentrated to a small volume to provide a crude extract. The concentrate of the ethanol phase was suspended in deionized water, successively partitioned with CHCl_3_, EtOAc, and *n*-BuOH and subjected to column chromatography over silica gel. The CHCl_3_ and EtOAc extracts were combined and subjected to column chromatography over silica gel eluting with a gradient CHCl_3_-MeOH (0- > 100%), followed by column chromatography over octadecyl silane and sephadex LH-20. Preparative thin layer chromatography was used to purify the compounds, and purity over 95% was verified by HPLC-UV. To characterize the compounds, UV and NMR data were collected from UV-2550 spectrometer and Bruker Avance 500 or 600 NMR spectrometers.

### 5.2. Cell Cultures

MT-4, U373-CD4-CXCR4, U373-CD4-CCR5, HeLa and TZM-Bl cell lines were obtained through the NIH AIDS Reagent Program. HEK 293, Vero, MDCK, and CRFK cells were purchased from ATCC (Manassas, VA, USA). HeLa-P4-CXCR4-LTRLacZ and Hela-ENV-Lai cells [26] were kindly given by Dr. Marc Alizon, Institute Pasteur, Paris. MT-4 cells were cultured in RPMI 1640 (Lonza, Wijchen, The Netherlands) containing 10% heat-inactivated fetal bovine serum (FBS) (Lonza, Netherlands) and 2mM L-glutamine (Invitrogen, Gosselies, Belgium). U373-CD4-CXCR4 and U373-CD4-CCR5 were cultured in RPMI 1640 containing 10% FBS, 2mM L-glutamine, 200 μg/mL geneticin (Invitrogen, Belgium), and 100 μg/mL hygromicin B (Invitrogen). HEK 293, Vero, Hela, and TZM-Bl were cultured in dulbecco’s modified eagle medium (DMEM) (Lonza) containing 10% FBS and 2mM L-glutamine. MDCK and CRFK cells were in eagle’s minimum essential medium (Lonza) containing 10% FBS. HeLa-P4-CXCR4-LTRLacZ cells were cultured in DMEM containing 10% FBS, 2mM L-glutamine, and 500 μg/mL geneticin. Hela-ENV-Lai cells were cultured in DMEM containing 10% FBS, 2mM L-glutamine, and 2 μM methotrexate (Sigma-Aldrich, Liège, Belgium).

### 5.3. Viral Infection with HIV-1 Reference Strains and Clinical Isolates

Peripheral blood mononuclear cells (PBMCs) were isolated from healthy donors’ buffy coats (Red Cross of Luxembourg, Luxembourg, Luxembourg) using Ficoll-Hypaque gradient as indicated previously (Sigma-Aldrich, Liège, Belgium). PBMCs were stimulated using 10 μg/mL phytohemagglutinin (PHA-P, Sigma Aldrich) for 48 h and recombinant IL-2 (10 U/mL, Roche, Sigma-Aldrich, Liège, Belgium) for another 24 h. Stimulated PBMCs were infected by the HIV-1 reference strains IIIB/ADA-M or primary clinical isolates expanded in culture from anonymized left-over samples (Centre Hospitalier de Luxembourg, Luxembourg, Luxembourg) in the presence or absence of drugs replaced every other day during 7 days. P24 production was measured in supernatants by ELISA (Perkin Elmer, Brussels, Belgium). Efavirenz (EFV) and azidothymidine (AZT) were obtained from Sigma_Aldrich. Enfuvirtide (T20) was purchased from Eurogentec (Seraing, Belgium).

### 5.4. Cytotoxicity and Apoptosis Assays

To assess drug cytotoxicity, PBMCs were incubated with or without drugs in a 96-well round bottom plate (Thermo Fisher, Asse, Belgium) at 2 × 10^5^/200 μL/well. After 2 days, cells were washed with PBS and stained with 0.1 μL near-IR fluorescent reactive dye (Life technologies, Ghent, Belgium) in 100 μL PBS for 20 min at room temperature in dark. Stained cells were measured by flow cytometry (FACSCanto, BD biosciences, Belgium).

To measure apoptosis, PBMCs were incubated with or without drugs in a 96-well round bottom plate at 2 × 10^5^/200 μL/well. After 2 days, cells were washed with PBS and stained with 2.5 μL Annexin-V-APC (Biosciences, Aalst, Belgium) in 100 μL staining buffer containing 10 mmol/L HEPES pH7.4, 140 mmol/L NaCl, and 2.5 mmol/L CaCl_2_ for 20 min at room temperature in dark. Cells were then washed with staining buffer and incubated with 100 μL 0.1 μg/mL propidium iodide (PI) (Thermo Fisher, Asse, Belgium) for 5 min at room temperature in dark. Stained cells were measured by flow cytometry (FACSCanto, BD, Aalst, Belgium).

### 5.5. Multi-Dosing Time Assay and Drug Combination Assay

For normal treatment, U373-CD4-CXCR4/U373-CD4-CCR5 cells were infected by pseudotyped virus pNL4.3Δ*Env*Luc-HXB2/pNL4.3Δ*Env*Luc-BAL [41], respectively, in the presence or absence of drugs through 2 h spinoculation at 1200× *g*. After infection, cells were then cultured in fresh culture medium for 48 h. Luciferase activity in cell lysates expressed as relative light units was measured via Luciferase System kit (Promega, Leiden, Netherlands).

In multi-dosing time assay, four different treatments were performed: 2 h pre-incubation of drugs on U373-CD4-CXCR4/U373-CD4-CCR5 cells, 2 h pre-incubation of drugs on pseudotyped virus pNL4.3Δ*Env*Luc-HXB2/pNL4.3Δ*Env*Luc-BAL, 2 h co-incubation of drugs, cells and viruses during spinoculation (normal treatment), and 2 h incubation of drugs on infected cells after spinoculation (post-infection).

In drug combination assay, U373-CD4-CXCR4/U373-CD4-CCR5 cells were co-incubated with CE or piceatannol combined with the CXCR4 inhibitor AMD3100 (Sigma, Belgium) or with the CCR5 inhibitor Maraviroc (Sigma, Belgium) or with the fusion inhibitor T20 during infection with the pseudotyped viruses by spinoculation as described above (normal treatment). The combination index (CI) was calculated at the EC_95_-level using CompuSyn software (ComboSyn, USA). According to Chou and Talalay’s method, CI > 1.1 means antagonism, CI < 0.9 means synergy, and 0.9 < CI < 1.1 means additive effect of the drugs.

### 5.6. Fusion Assay

Fusion inhibition was evaluated as previously described [26]. Briefly, HeLa-ENV-Lai cells were pre-incubated with or without drugs for 2 h and added to HeLa-P4-CXCR4-LTRLacZ cells which were placed in a 96-well plate 1 day before. After 24 h, cells were washed with PBS and then incubated with 50 μL containing 0.5% NP-40 (Sigma, Belgium) for 15 min at room temperature. Fifty microliters chlorophenol red-β-D-galactopyranoside (CPRG) reagent (Roche, Sigma-Aldrich, Liège, Belgium) was then added into the plate. After 30 min incubation at room temperature in dark, β−galactosidase was assessed by measuring OD_562_ of the cells via POLARstar Omega plate reader (BMG Labtech, Ortenberg, Germany).

### 5.7. Broad Spectrum Antiviral Activity

Antiviral assays were previously described [27] for the following viruses: HSV-1 (KOS), HSV-2 G, HSV-1 TK KOS ACV; VSV, coxsackie virus B4, RSV, Coxsackie virus B4, para-influenza-3 virus, reovirus-1, sindbisvirus, punta toro virus, yellow fever virus, influenza A/H1N1 A/Ned/378/05, influenza A/H3N2 A/HK/7/87, influenza B B/Ned/537/05; human corona virus, feline corona virus, feline herpes virus, and HCV (Jc1).

### 5.8. CD4-gp120 Interaction Assay

Human soluble CD4 (sCD4) was immobilized in a 96-well plate by adding 100 ng sCD4 (R&D Systems, Belgium) in 100 μL PBS per well and incubated at 4 °C. After 24 h, the CD4 coated plate was washed with 1% BSA-PBS and blocked by adding 100μL 5% BSA-PBS per well for 1 h at 4 °C. The coated plate was then washed and incubated with or without drugs for 1 h at 4 °C. After washing, 100 ng HIV-1 gp120 protein (Fitzgerald, Acton, MA, USA) was added into the plate and kept for 1 h at 4 °C. The plate was washed, and anti-HIV-1 gp120 (Aalto Bio Reagents, Dublin, Ireland) (100 ng/100 μL PBS) was added. After 1 h, the plate was washed and anti-sheep HRP (Sigma, Belgium) (100 ng/100 μL PBS) was added. After 1 h incubation at 4 °C, 100 μL o-phenylenediamine dihydrochloride (OPD) (Thermo Fisher, Asse, Belgium) was added and incubated for 20 min in dark. One hundred microliters 0.5M H_2_SO_4_ was finally added to stop the reaction. OD_492_ and OD_630_ were measured by POLARstart Omega Plate Reader. OD_492_ − OD_630_ was calculated.

### 5.9. Binding Assays and Co-Receptor Internalization

Binding competition between increasing concentrations of compounds and FITC-conjugated mouse anti-human CD4 clone RPA-4 (Biolegend, Amsterdam, Netherlands), PE-conjugated mouse anti-Human CXCR4 clone 12G5, and PE-conjugated mouse anti-human CCR5 clone 2D7 (BD Pharma, Aalst, Belgium) to U373-CD4-CXCR4 and U373-CD4-CCR5 cells was first tested by flow cytometry (FACSCanto). The chemokines CXCL12 and CCL5 (50 nM) (Peprotech, London, United Kingdom) were used as positive controls, and near-IR fluorescent reactive dye was added simultaneously to evaluate cell viability. After 1 h of incubation at 4 °C, cells were washed with FACS buffer (1% bovine serum albumin, 0.1% N3Na in PBS, Sigma), and Mean Fluorescence Intensity (MFI) was measured. To study co-receptor internalization, compounds were incubated alone (agonist mode) or in the presence of 50 nM chemokines (CXCL12/CCL5) (antagonist mode) with MT-4 and U373-CD4-CCR5 cells at 37 °C for 1 h. Internalization was stopped after 30 min by addition of NaN_3_ (0.1%) on ice. Cells were stained with anti-human CXCR4 clone 4G10 (BD, Belgium) or anti-human CCR5 (CD195) clone T21/8, (eBioscience, Asse, Belgium), Near-IR fluorescent reactive dye during 1 h at 4 °C, washed with FACS buffer, and a secondary PE-conjugated goat anti-mouse antibody (Jackson ImmunoResearch, West Grove, PA, USA).

### 5.10. Dual-Chamber and DC-SIGN Transmission Assays

Dual chamber transmission assay was performed as previously described [28]. HeLa cells were seeded into an upper chamber of a transwell plate (Sigma-Aldrich, Liège, Belgium), while TZM-bl cells were seeded in the lower chamber. Trans Epithelial Electric Resistance (TEER) was measured using Millicell-ERS Volt-Ohm Meter. HIV-1 ADA-M (200 pg) and drugs were added to the upper chamber after 4 days when TEER reached 150 Ohm/cm^2^. 24 h after infection luciferase value of TZM-bl cells lysate was measured using the Luciferase System Kit and the POLARstar Omega Plate Reader. Data were analyzed using GraphPrism. The HIV-1 DC-SIGN transmission assay was performed as previously described [27]. To investigate the cellular activation induced by CE or piceatannol, CD25 and CD69 expression was measured on PBMCs after incubation with CE/piceatannol or 10 µg/mL PHA-P for 24 h at 37 °C using FITC-conjugated anti-CD4, PE/Cy7-conjugated anti-CD25, PE-conjugated anti-CD69 mAbs (Biolegend, Amsterdam, Netherlands), and the near-IR fluorescent reactive dye.

### 5.11. In Silico Ligand-Based Study

For SAR purposes, molecules **1**–**11** were purchased from MolPort (Riga, Latvia), while molecules **12**–**17** (purity > 95% by HPLC) were retrieved from an in-house library of natural products previously characterized [29,42]. To provide insights into the mechanism of action of bioactive hits, a ligand-based study was conducted using the ROCS program (Openeye Scientific Software) version 3.3.0.3 [43]. A query was built on the chemical structure of piceatannol and used to screen the conformational database of compounds **1**-**17** that was generated by OMEGA (OpenEye Scientific Software) version 3.1.0.3 [44] using default settings. Ligands similarity was assessed by the TanimotoCombo scores, while normalized similarity scores were calculated by dividing the TanimotoCombo value by two. All the possible stereochemical configurations of cassiabrevone and 4′7-Dihydroxyflavan (4,6)-3, 4´, 5,7- tetrahydroxyflavan were analyzed; the reported normalized similarity and TanimotoCombo scores refer to the isomers endowed with the highest similarity to piceatannol.

## Figures and Tables

**Table 1:** Evaluation of CE and piceatannol’s activity against various viruses.

Cells	Viruses	CE (µg/mL)	Piceatannol(µM)
HEL	Herpes simplex virus-1 (KOS)	46.7 ± 2.9	47.5 ± 3.5
HEL	Herpes simplex virus-2 (G)	39.5 ± 5.5	45.0 ± 1.8
HEL	Herpes simplex virus-1 TK KOS ACV	45.0 ± 2.6	45.4 ± 4.0
U87	Vesicular stomatitis virus	>100	79.2± 17
MDCK	Influenza A/H1N1 A/Ned/378/05	>100	>100
MDCK	Influenza A/H3N2 A/HK/7/87	>100	>100
MDCK	Influenza B B/Ned/537/05	>100	>100
Huh 7-D	Hepatitis C virus (Jc1)	>100	>100
HeLa	Coxsackie virus B4	>100	>100
vero	Coxsackie virus B4	>100	>100
HeLa	Respiratory syncytial virus	>100	>100
vero	Para-influenza-3 virus	>100	>100
vero	Reovirus-1	>100	>100
vero	sindbisvirus	>100	>100
vero	Punta toro virus	>100	>100
vero	Yellow fever virus	>100	>100
CRFK	Feline corona virus (FIPV)	>100	>100
CRFK	Feline herpes virus	>100	>100
HEL	Human corona virus	>100	>100

IC_50_ (inhibitory concentration of viral replication by 50%), CE, crude extract of *C. abbreviata*.

## Data Availability

All the data are contained within the article.
